# Differences in rehabilitation for high-risk newborns: The impact of neonatal intensive care unit hospitalization

**DOI:** 10.1371/journal.pone.0322998

**Published:** 2025-05-09

**Authors:** Kyoung Yee Kang, Eun Sil Kang, Hye-Kang Park, Seung Been Hong, Ha Lim Lee

**Affiliations:** 1 Department of Children’s Rehabilitation, Public Children’s Rehabilitation Hospital, National Health Insurance Service Ilsan Hospital, Goyang, Gyeonggi-do, Republic of Korea; 2 Department of Physical Medicine and Rehabilitation, National Health Insurance Service Ilsan Hospital, Goyang, Republic of Korea; 3 Department of Physical Medicine and Rehabilitation, Rehabilitation Center, National Health Insurance Service Ilsan Hospital, Goyang, Gyeonggi-do, Republic of Korea; Cairo University / Jouf University, SAUDI ARABIA

## Abstract

There is growing recognition of the importance of rehabilitation through immediate and long-term follow-up, and neonatal intensive care unit (NICU) aftercare is emerging as an important field to consider rehabilitation services. An increasing number of children born are admitted to the NICU with complications commonly related to low birth weight, premature birth, and development of underlying diseases. Early initiation of rehabilitation services in the NICU has become more common and includes therapies relating to feeding tube removal and pulmonary breathing. We investigated the patterns of rehabilitation utilization (rehabilitation frequency, moving to an area for rehabilitation treatment) and medical expenses based on NICU hospitalization history. Data from the Korean National Health Insurance Service Database over a span of ten years were reviewed, with an observation period of 3 years after the first rehabilitation session. The newborns were divided into two groups: 16,626 in the NICU group and the non-NICU group, matched 1:1 based on NICU hospitalization history. The number of rehabilitation treatments in the non-NICU group was significantly higher over the two years following the initial rehabilitation session (*p* < 0.05). In contrast, the total medical expenses during the 6 months following the initial rehabilitation session were more than six times higher in the NICU group (KRW 1,868,516 vs. 11,348,940, *p* < 0.0001). The NICU group showed significantly more discrepancies between their residence and the first rehabilitation treatment area (9.5% vs. 13.4%, *p* < 0.001). Results indicate that the amount of rehabilitation sessions and access to rehabilitation for individuals with a NICU history is lower compared to those without a NICU history. Therefore, national support is needed to revitalize rehabilitation procedures and reduce medical expenses in the NICU group, and further studies should focus on novel methods to revitalize NICU rehabilitation.

## Introduction

High-risk newborns are infants who experience difficulties adapting after birth, have an elevated risk of mortality and morbidity, and require immediate medical attention in the neonatal intensive care unit (NICU) [1]. An increasing percentage of newborns (10%–15%) require admission to the NICU [2]. Most hospitalizations in the NICU are caused by low birth weight or premature birth, however, the rate of NICU use for babies born at ≥34 weeks has recently increased due of the development of underlying diseases [3,4]. As more newborns require NICU hospitalization, families are facing an increasing burden of medical expenses [5,6]. After discharge from the NICU, 53% of parents were worried about healthcare costs, and 78% discussed or received supplemental security income [5]. These costs are associated with treatment during NICU hospitalization and after discharge, with rehabilitation treatment contributing significantly [7,8]. As high-risk newborns in the NICU are at risk of developing neurodevelopmental morbidities, rehabilitation efforts are increasing through ICU aftercare, including both early follow-up during hospitalization and late follow-up after discharge [9,10]. Lambiase et al. [11] reported that infants admitted to the NICU had more medical visits and hospitalizations due to developmental issues during the first year after discharge compared to normal infants. Additionally, without the support of the national insurance programs, the monthly cost of private rehabilitation treatment can create a financial burden for parents [11,12]. In Korea, all citizens are required to enroll in the national health insurance system that provides universal coverage, significantly reducing medical expenses for patients. Therefore, researchers can examine the details of medical utilization benefits by utilizing health insurance data. However, there is a lack of research regarding rehabilitation treatment and associated medical expenses in Korean NICUs.

Rehabilitation services, including physical therapy (PT), occupational therapy (OT), and speech therapy (ST), are commonly provided in the NICU [13]. Most patients in the NICU received OT more frequently than PT, whereas ST was provided to approximately half of hospitalized patients [14]. In Korea and Canada, feeding or dysphagia treatment is performed by occupational therapists, and there have been cases with no STs included in the rehabilitation treatment team at general hospitals [14,15]. In contrast, according to a study based on the Korean National Health Insurance database, patients with prolonged ICU stays received frequent rehabilitation [16]. Rehabilitation treatment is continuously needed as muscle strength and functional abilities decrease as the ICU length of stay (LOS) increases and it is important to reduce the ICU LOS through early rehabilitation in the acute phase of hospitalization [16,17]. For patients with neurological disorders, PT should begin as early as the third day after ICU admission and should include rehabilitation that combines positive sensory experiences and motor training at least three times a week [18,19]. Early rehabilitation in the NICU can lead to reduced oxygen supplementation and gastric tube use, as well as shorter hospitalization for infants with stable respiration and oxyhemoglobin saturation under non-invasive ventilation, compared to a control group receiving no intervention [20]. Thus, timely initiation of rehabilitation in the NICU is of utmost importance as its effects persist even after discharge [21]. However, most NICU-related medical facilities are concentrated in large cities, leading to a significant lack of available medical services during hospitalization and after discharge, depending on the region [22–24]. Geographic inequities can cause families to move regions for rehabilitation services, creating extra financial and logistical burdens [23]. Regional gaps in medical service availability not only impacts the frequency and quality of rehabilitation, but also affects long-term developmental outcomes for high-risk newborns [24]. However, there is a lack of knowledge on the regional differences in rehabilitation treatment for NICU patients.

Therefore, this study aimed to compare rehabilitation treatment patterns depending on whether patients were admitted to the NICU. We hypothesized that the number of rehabilitation sessions, consistency between residence and first rehabilitation area, and medical expenses are influenced by NICU admission history.

## Materials and methods

### Study population

**Fig 1:**
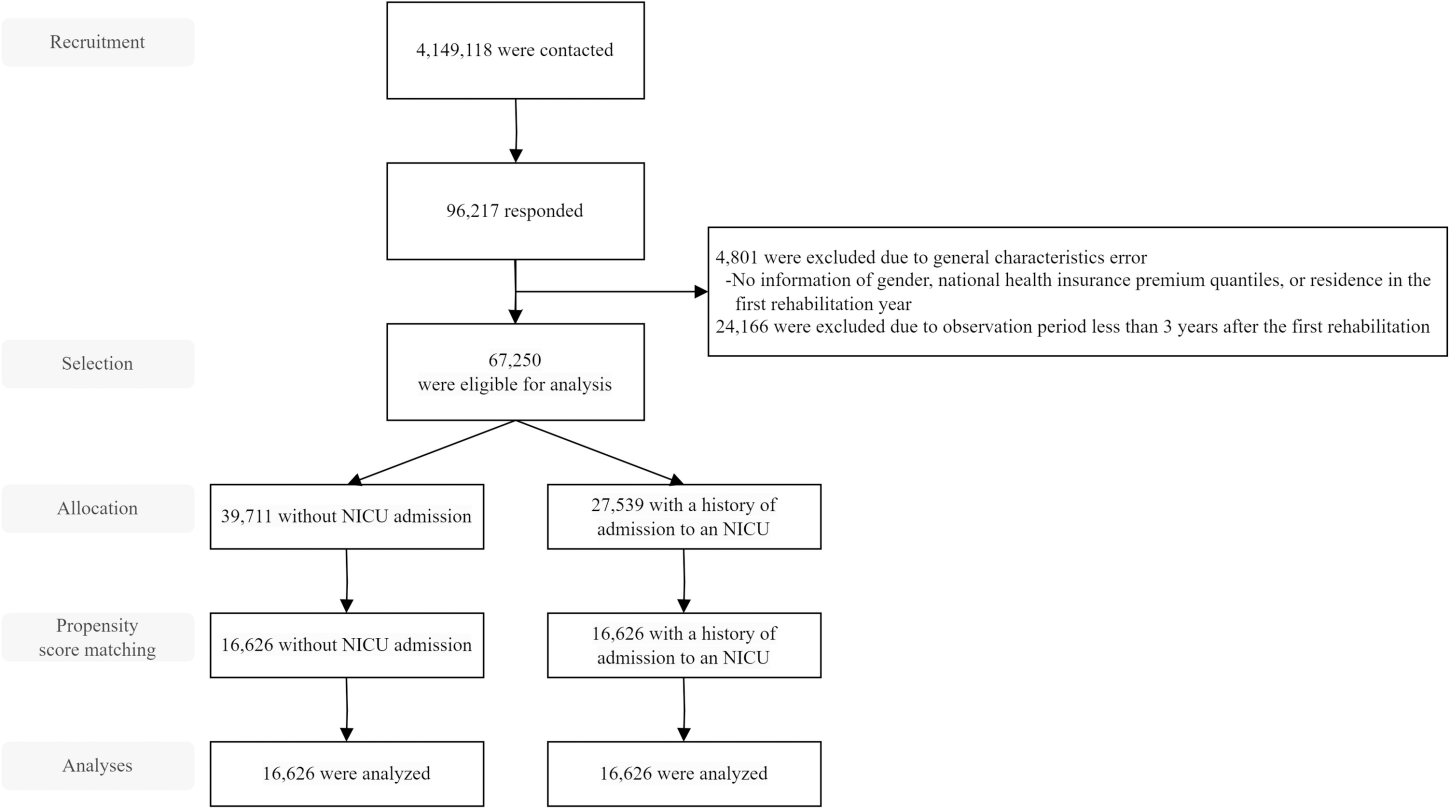
Flow of participant selection. NICU: neonatal intensive care unit.

A nationwide retrospective cohort analysis was performed using claims data provided by the Korean National Health Insurance Service (NHIS) database for infants born between January 1, 2011 and December 31, 2021. The use of claims data was approved by the NHIS (NHIS-2023-1-244), and pseudonymized data were provided to prevent personal information identification. The inclusion criterion were patients with a history of rehabilitation, and the exclusion criteria were 1) patients with errors in general characteristics (no sex, no insurance quantile, and no residence information in the first year of rehabilitation year); 2) an observation period less than three years after the first rehabilitation session; 3) any missing data during the three year follow-up period. Missing data included the absence of values for the number of rehabilitation sessions and medical costs during the follow-up period. The 3-year follow-up period is critical in clinical practice for the identification of developmental disabilities, including cerebral palsy, as well as for the monitoring of respiratory complications and infections, underscoring the necessity for early intervention and comprehensive management during this developmental stage [25–27]. Therefore, this study set a 3-year follow-up period to examine whether the 3-year rehabilitation treatment was being carried out appropriately. Data of 67,250 newborns were included in this study, and for subsequent analysis, patients were divided into two groups based on being admitted (NICU) or not admitted (non-NICU) to the NICU. The analysis was conducted using 1:1 propensity score matching (PSM) on a total of 33, 252 selected targets (Fig 1).

This study was approved by the Institutional Review Board of the National Health Insurance Service Ilsan Hospital (IRB No. NHIMC-2022-10-018).

### Definitions

Rehabilitation referred to the behavior provided by the therapist based on the claim code, and the NHIS claims codes used in this study are shown in S1 Table. In this study, claim codes for rehabilitation treatment included codes for exercise and functional electrical stimulation aimed at restoring central nervous system, as well as activities of daily living training. Codes related to cardiopulmonary rehabilitation, pain treatment, and skin-related treatment were excluded. PT was defined as one of six types of therapy that occurred. A complex therapeutic exercise involves muscle strengthening and functional training using equipment (30 minutes per session). Rehabilitative development therapy (30 minutes per session), is a personalized approach that focuses on muscle strengthening, sensory integration, and myofascial release techniques based on neurodevelopmental therapy (NDT) and is provided by a physical therapist trained in a specialized course of over 120 hours. Functional electrical stimulation therapy (20 minutes per session), involved strengthening muscle strength, preventing muscle atrophy, and retraining. Rehabilitative functional training (30 minutes per session) involves mobilization training and gait training provided by physical therapists. Aquatic therapy was defined as whole-body pool therapy (30 minutes per session) provided in water by a physical therapist to conduct various motion training using limb movement using equipment such as pool floats.

OT included complex OT (10-30 minutes per session) or special OT (30 minutes per session), and activities of daily living training (20 minutes per session) as provided by occupational therapists. The complex OT and special OT are both treated to enhance the patient’s function, with the main difference is the duration of sessions. Activities of daily living training include skills such as eating, dressing and undressing, toileting, and hygiene. Dysphagia therapy was defined as rehabilitative dysphagia therapy (30 minutes per session) and functional electrical stimulation for rehabilitative dysphagia therapy (30 minutes per session) provided by occupational therapists.

Patients with a history of rehabilitation refers to those who receive PT or OT services at least once during a 3-year observation period. Age at the first rehabilitation session was determined by subtracting the date of birth from the index date of the claim and then dividing the difference in 1-year increments, ranging from 0 to 4 years or older. Income (measured in Korean won [KRW]) referred to the quintile of insurance premiums and was divided into the 1st–5th, 6th–10th, 11th–15th, and 16th–20th quintiles of medical benefits. Total medical expenses (in KRW) were defined as the sum of the insurer contributions and out-of-pocket expenses.

The disease of this study was defined as Disease of Interest five, based on previous studies among a total of sixteen diseases recognized by the Korea Insurance Corporation for rehabilitation treatment fees and included the Korean Standard Classification of Diseases major or injured disease codes: **1)** “Cerebral palsy and other paralytic syndromes” (G80-83) 2) “Lack of expected normal physiological development” (R62) **3)** “Certain conditions originating in the perinatal period” (P00-96) without “Disorders related to short gestation and low birth weight” (P07) **4)** “Congenital malformations, deformations and chromosomal abnormalities” (Q00-96), and **5)** “Epilepsy and status epilepticus” (G40-41). Except for the five diseases of interest, the remaining diseases are categorized as **6)** “other diseases,” including “Malignant neoplasm of brain” (C71), “Inflammatory diseases of the central nervous system” (G00-G09), “Systemic atrophies primarily affecting the central nervous system” (G10-12), “Extrapyramidal and movement disorders” (G20-G26), “Demyelinating diseases of the central nervous system (G35-G37), “Nerve, nerve root and plexus disorders” (G50-G59), “Polyneuropathies and other disorders of the peripheral nervous system” (G60-G64), “Diseases of myoneural junction and muscle” (G70-G73), “Other disorders of the nervous system” (G90-G99), “Cerebrovascular diseases” (I60-I69),and “Sequelae of injures, of poisoning and of other consequences of external causes” (T90-T98) [28,29].

Korea is divided into administrative districts consisting of cities and provinces, totaling 17 regions. Based on criteria from previous research, the top six regions (Seoul, Busan, Daegu, Incheon, Daejeon, and Gyeonggi) with the highest number of rehabilitation treatment claims, according to health insurance data, are classified as rehabilitation invulnerable areas. The remaining regions are categorized as vulnerable areas [16].

### Statistical analysis

Prior to statistical analysis, PSM was conducted to ensure the equivalence of diagnoses among infants with a history of rehabilitation and those with and without a history of NICU hospitalization born between 2011 and 2021. PSM can ensure the homogeneity of groups by correcting for selection bias, a limitation of observational studies, and adjusting for covariates that may influence the results [30]. Before constructing the matching data, logistic regression analysis was performed to calculate the propensity score. Covariates included age, sex, residential group, income, and disease status, all of which are potential confounders that could affect both groups. One-to-one matching and nearest-neighbor matching were employed; both approaches do not allow for duplication. A caliper of 0.2 was applied to ensure that matched pairs were similar enough for meaningful comparison [31]. The caliper size restricts the matching procedure to avoid poor matches by ensuring a minimal difference in propensity scores between the NICU group and non-NICU group. Sensitivity analysis, which considered the ratio of control participants, was performed to confirm robustness of matching (S2 Table); this analysis ensured that the conclusions drawn from the matched data were not unduly influenced by the specific matching choices made.

Frequencies and ratios were presented as an analysis method for the study participants’ demographic characteristics, socioeconomic characteristics, and types of rehabilitation, and the difference in ratios between the two groups was tested using chi-square values. Additionally, the mean and standard deviation of age, medical expenses, and number of rehabilitation treatments after the first rehabilitation session were calculated, and the mean difference between the two groups was tested using a t-test. All statistical analyses were performed with SAS version 9.4 (SAS Institute, Cary, NC, USA), and *p* < 0.05 were considered statistically significant.

## Results

Among the 67,250 eligible patients, 39,711 (59.0%) and 27,539 (41.0%) were classified into the non-NICU and NICU groups, respectively. PSM resulted in a total of 16,262 patients per group (Table 1).

**Table 1:** Demographic characteristics.

Variables		Before matching			After matching		
		Non-NICU (n = 39,711)	NICU (n = 27,539)	*p*-value	Non-NICU (n = 16,626)	NICU (n = 16,626)	*p*-value
**Age of first rehabilitation,** **years**	0	24,444 (61.6%)	23,643 (85.9%)	<0.0001	13,217 (79.5%)	13,217 (79.5%)	1.0000
	1	5,782 (14.6%)	2,564 (9.3%)		2,240 (13.5%)	2,240 (13.5%)	
	2	2,791 (7.0%)	597 (2.2%)		533 (3.2%)	533 (3.2%)	
	3	2,516 (6.3%)	366 (1.3%)		307 (1.8%)	307 (1.8%)	
	≥4	4,178 (10.5%)	369 (1.3%)		329 (2.0%)	329 (2.0%)	
**Sex**	Male	23,143 (58.3%)	15,530 (56.4%)	<0.0001	9,581 (57.6%)	9,581 (57.6%)	1.0000
	Female	16,568 (41.7%)	12,009 (43.6%)		7,045 (42.4%)	7,045 (42.4%)	
**Region**	invulnerable area	27,062 (68.1%)	18,565 (67.4%)	0.0451	11,587 (69.7%)	11,587 (69.7%)	1.0000
	vulnerable area	12,649 (31.9%)	8,974 (32.6%)		5,039 (30.3%)	5,039 (30.3%)	
**Income level** ^**a**^	Medical aids, 1st–5th income group	4,314 (10.9%)	3,180 (11.5%)	<0.0001	1,799 (10.8%)	1,799 (10.8%)	1.0000
	6th–10th income group	6,735 (17.0%)	4,964 (18.0%)		2,852 (17.2%)	2,852 (17.2%)	
	11th–15th income group	15,456 (38.9%)	10,645 (38.7%)		6,558 (39.4%)	6,558 (39.4%)	
	16th–20th income group	13,206 (33.3%)	8,750 (31.8%)		5,417 (32.6%)	5,417 (32.6%)	
**Disease** ^**b**^	G40-41	4,576 (11.5%)	4,570 (16.6%)	<0.0001	2,054 (12.4%)	2,054 (12.4%)	1.0000
	G80-83	7,004 (17.6%)	9,419 (34.2%)	<0.0001	3,772 (22.7%)	3,772 (22.7%)	1.0000
	P00-96 (without P07)	24,632 (62.0%)	26,582 (96.5%)	<0.0001	15,720 (94.6%)	15,720 (94.6%)	1.0000
	Q00-99	23,997 (60.4%)	20,504 (74.5%)	<0.0001	11,659 (70.1%)	11,659 (70.1%)	1.0000
	R62	20,796 (52.4%)	20,009 (72.7%)	<0.0001	9,977 (60.0%)	9,977 (60.0%)	1.0000
	Other disease	9,486 (23.9%)	8,405 (30.5%)	<0.0001	4,270 (25.7%)	4,270 (25.7%)	1.0000

All values are presented as frequency (%).

^a^lower group number indicates lower income, and medical aid is classified as a public medical insurance system where the government supports medical expenses for low-income individuals.

^b^Based on Korean Standard Classification of Diseases codes: G40-41 = Epilepsy and status epilepticus, G80-83 = Cerebral palsy and other paralytic syndromes, P00-96 (without P07) = Certain conditions originating in the perinatal period (without Disorders related to short gestation and low birth weight), Q00-96 = Congenital malformations, deformations and chromosomal abnormalities, R62 = Lack of expected normal physiological development.

NICU, neonatal intensive care unit.

The start of rehabilitation was considered if at least one PT, OT, aquatic therapy, or dysphagia therapy was provided. The mean age during the first rehabilitation session in both groups was significantly lower in the NICU group than in the non-NICU group (0.58 years vs. 1.43 years; *p* < 0.001).

The total number of rehabilitation treatments per patient was significantly higher in the non-NICU group than in the NICU group for 2 years after the first rehabilitation session (*p* < 0.05). In particular, during the 6 months following the first rehabilitation session, the total number of rehabilitation treatments was 27.5% lower in the NICU group compared to the non-NICU group (*p* < 0.0001). Additionally, PT sessions were significantly more frequent in the non-NICU group than in the NICU group for 1.5 years after the first rehabilitation session (*p* < 0.0001). Aquatic therapy showed no significant differences in any period after the first rehabilitation session.

OT sessions were significantly more frequent in the non-NICU group than in the NICU group for 2.5 years after the first rehabilitation session (*p* < 0.05). Dysphagia therapy was significantly more frequent in the non-NICU group than in the NICU group for one year after the first rehabilitation session (*p* < 0.0001; Table 2).

**Table 2:** Number of rehabilitation sessions per person within 6 months after the first rehabilitation session.

	0–0.5 years		0.5–1.0 year		1.0–1.5 years		1.5–2 years		2.0–2.5 years		2.5–3.0 years	
	Non-NICU	NICU	Non-NICU	NICU	Non-NICU	NICU	Non-NICU	NICU	Non-NICU	NICU	Non-NICU	NICU
Total rehabilitation	30.2 ± 47.6 (16,626)	21.9 ± 39.9*** (16,626)	68.7 ± 88.1 (6,039)	55.7 ± 73.6*** (6,984)	91.1 ± 103.1 (4,154)	79.9 ± 94.2*** (4,879)	100.0 ± 110.7 (3,441)	93.6 ± 107.7* (3,802)	104.6 ± 113.0 (3,001)	102.3 ± 114.7 (3,220)	103.4 ± 110.9 (2,743)	102.6 ± 113.7 (2,909)
Physical therapy	24.0 ± 33.1 (15,754)	20.9 ± 30.2*** (12,064)	45.7 ± 55.6 (5,622)	37.9 ± 47.4*** (6,545)	58.5 ± 64.4 (3,655)	52.1 ± 59.5*** (4,425)	63.6 ± 68.3 (2,936)	60.3 ± 67.0 (3,294)	66.9 ± 70.2 (2,494)	66.9 ± 71.5 (2,676)	65.9 ± 68.5 (2,231)	65.6 ± 69.5 (2,419)
Aquatic therapy	5.2 ± 7.0 (122)	4.8 ± 9.0 (82)	10.0 ± 11.6 (150)	8.3 ± 12.0 (105)	11.3 ± 13.3 (178)	10.5 ± 12.1 (134)	13.2 ± 14.3 (182)	11.5 ± 12.1 (155)	12.6 ± 13.1 (167)	11.1 ± 12.5 (148)	15.6 ± 14.4 (149)	12.7 ± 13.8 (138)
Occupational therapy	25.5 ± 29.8 (4,558)	16.9 ± 24.7*** (5,761)	40.9 ± 38.5 (3,565)	33.3 ± 34.0*** (3,857)	47.1 ± 42.3 (3,175)	41.7 ± 39.3*** (3,452)	49.5 ± 44.6 (2,847)	46.0 ± 43.0** (3,027)	51.2 ± 44.7 (2,560)	48.6 ± 45.3* (2,730)	51.1 ± 43.9 (2,373)	49.7 ± 44.5 (2,458)
Dysphagia therapy	7.2 ± 12.3 (1,044)	2.7 ± 6.0*** (4,748)	14.1 ± 20.7 (779)	12.6 ± 18.1*** (960)	18.0 ± 23.4 (730)	16.6 ± 20.8 (861)	20.9 ± 29.0 (666)	20.4 ± 27.3 (789)	23.3 ± 30.5 (587)	23.4 ± 30.5 (684)	24.0 ± 29.6 (540)	25.0 ± 32.1 (631)

All values are presented as means±standard deviation (n). *P < 0.05; **P < 0.01; ***P < 0.001. NICU, neonatal intensive care unit.

**Fig 2:**
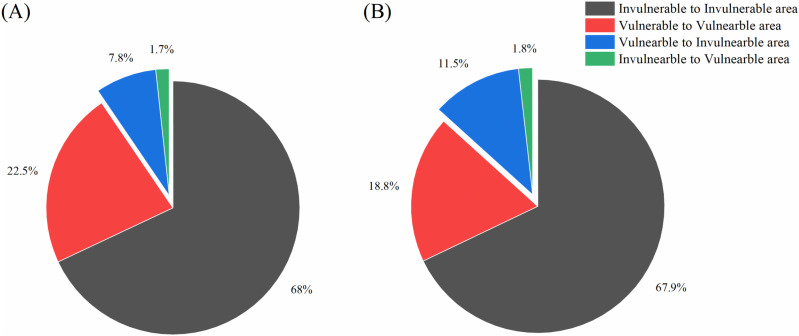
Regional movement at the time of the first rehabilitation. Blue and green in the pie chart represent discrepancies between the residential area and the first rehabilitation treatment area, while red and gray represent location agreement for the two events.

In this study, regional movement was determined by the subject’s place of residence and the location of their initial rehabilitation treatment service. If these two areas did not align, it indicated that the subject moved to a different region. There was a significant difference in regional movement for the first rehabilitation session, with 9.5% and 13.4% of patients in the non-NICU and NICU groups, respectively, traveling to different regions to undergo the first rehabilitation session (*p* < 0.0001). The residential area and the first rehabilitation treatment area were further classified as vulnerable and invulnerable areas. Regional movement wherein the patient’s residence was in a vulnerable area and the first rehabilitation session was in an invulnerable area, occurred in 7.8% of non-NICU cases and 11.5% of NICU cases. Regional movement of patients that resided in an invulnerable area with the first rehabilitation session in a vulnerable area occurred in 1.7% and 1.8% of the non-NICU and NICU group, respectively (Fig 2).

**Fig 3:**
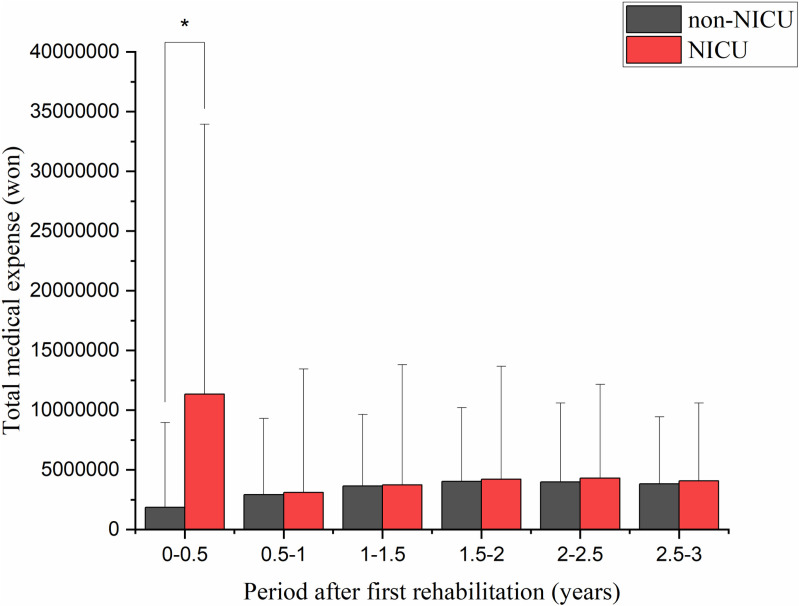
Total medical expenses after the first rehabilitation. Total medical expenses were significantly higher in the NICU group only for 6 months after the first rehabilitation session. NICU: neonatal intensive care unit.

**Fig 4:**
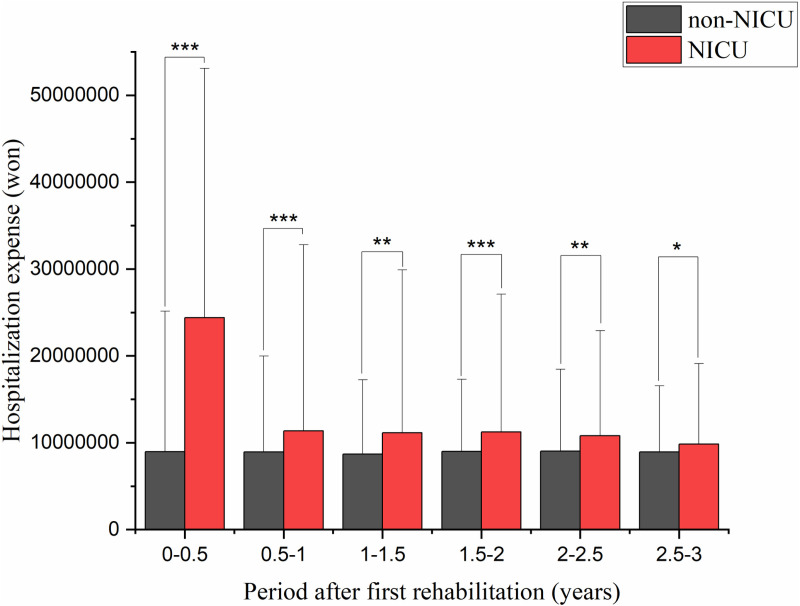
Hospitalization expenses after the first rehabilitation. Hospitalization expenses were significantly higher in the NICU group for 3 years after the first rehabilitation session, with the largest difference in the first 6 months. NICU: neonatal intensive care unit.

**Fig 5:**
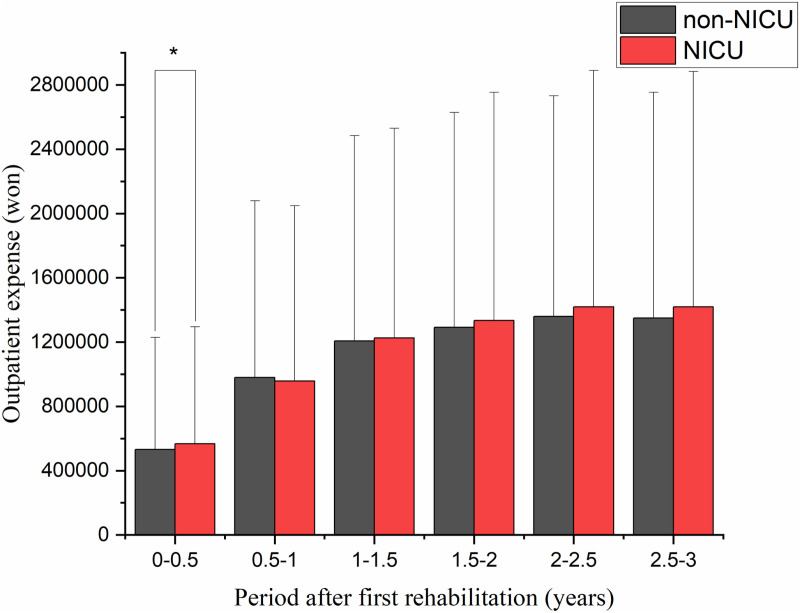
Outpatient expenses after the first rehabilitation. Outpatient expenses were significantly higher in the NICU group for 6 months after the first rehabilitation session. NICU: neonatal intensive care unit.

Based on medical bills that included rehabilitation charges after the first rehabilitation session, there was a significant difference in the total medical expenses per patient (KRW 1,868,516 for the non-NICU group and KRW 11,348,940 for the NICU group) for 6 months (*p* < 0.0001; Fig 3). Regarding hospitalization expenses, in the 7 months following the first rehabilitation session, costs were significantly greater in the NICU group compared with expenses in the non-NICU group (KRW 24,396,718 vs. KRW 8,955,365; *p* < 0.001). Moreover, hospital expenses in the NICU group were significantly greater than in the non-NICU group for 3 years after the first session (*p* < 0.05; Fig 4). For outpatient expenses, there was a significant difference between the non-NICU and NICU groups for 6 months after the first rehabilitation session (KRW 531,883 vs. KRW 566,593; *p* < 0.0001; Fig 5).

## Discussion

This study, which analyzed the rehabilitation patterns of newborns admitted and not admitted to the NICU, revealed that (1) after the first rehabilitation session, patients in the non-NICU group received a higher number of interventions during the subsequent two years, (2) there was a large discrepancy between the location of residence and the first rehabilitation among patients in the NICU group, and (3) the total expenses for both PT and OT within 3 years after the initial rehabilitation were higher in the NICU group.

Rehabilitation in the NICU can be categorized into behavioral organization, developmental interventions, feeding, handling and activity tolerance, neurodevelopment, oral motor, parent and team involvement, positioning, range of motion, and sensory-motor activities [13]. Although neonatal rehabilitation requires a team of physical, occupational, and speech therapists for a multidisciplinary approach, most university hospital NICUs in Korea do not have a separate rehabilitation team, and neonatal rehabilitation is mainly performed through PT and OT [32,33]. The role of speech therapists is important; however, occupational therapists perform therapy for dysphagia due to systemic limitations such as the lack of insurance benefits for speech therapy within the NICU and an insufficient number of speech therapists [15,34]. NICU physical therapists design, model, and coach positioning strategies to promote skeletal integrity, postural control, and sensorimotor development [35,36]. Early physiotherapy can help neonates prevent complications like respiratory distress syndrome, achieve motor milestones, and reduce irritability [37]. Children requiring mechanical ventilation or those with neuromotor impairments can improve bodily functions and activity levels through aquatic therapy [38,39]. Additionally, for neonates, aquatic therapy can help normalize muscle tone, strengthen active movement, and improve postural organization [40]. Occupational therapists play a crucial role in promoting the self-regulation of infants, interacting with caregivers, and feeding and swallowing skills in acute care settings, including in NICUs [41,42]. Swallowing rehabilitation in newborns is important for nutritional requirements, body growth, feeding activities, and prevention of choking and aspiration pneumonia [43]. Therefore, this study examined rehabilitation according to NICU admission by evaluating patients according to whether they received physical, aquatic, occupational, and dysphagia therapies.

According to a study conducted by Ross et al. [13], rehabilitation for infants in the NICU usually starts between 30.3 and 35.9 weeks after postmenstrual age and is carried out 1.1–1.8 times per week until the infant is discharged from the NICU. In this study, patients who had previously been hospitalized in the NICU underwent their first rehabilitation session at approximately 7 months of age, on average. Following this initial intervention, the frequency of rehabilitation sessions gradually increased from an average of 21.9 sessions within 6 months after the first rehabilitation session to 102.6 sessions in 2.5–3.0 years after the first rehabilitation session. However, compared with the non-NICU group, the number of rehabilitation sessions within 2 years after the first rehabilitation session was significantly lower. A low rehabilitation rate among children under 3 years old admitted to the pediatric ICU may be attributed to challenges such as the necessity of mechanical ventilation due to severe illness, as noted in previous research [16]. Additionally, patients in the ICU have limitations in receiving rehabilitation due to the lack of medical personnel and education related to ICU rehabilitation [44]. The current status of PT in the NICU in Korea is similar, with most hospitals having one physical therapist in charge of the NICU and this therapist treating no more than five patients for less than five hours a week [15]. In this study, it is believed that patients with a history of NICU hospitalization were not provided opportunities for rehabilitation because of factors that hindered the availability of rehabilitation sessions. The absence of rehabilitation treatment can adversely affect ICU patients’ physical function, recovery speed, and the efficient use of hospital resources [44]. Nonetheless, the relationship between neonatal rehabilitation and neurobehavioral outcomes is unclear due to various factors, including early discharge, medical stability, and complexities associated with medically fragile infants, which complicate the establishment of standard care protocols [13]. Additionally, this study highlighted the difference in the number of rehabilitation therapy sessions between the non-NICU and NICU groups but did not analyze variables that demonstrate clinical significance. Follow-up studies should further investigate the relationship between the number of rehabilitation treatments received by NICU patients and their clinical outcomes. Additionally, ongoing efforts are needed to establish standards for optimal care in this unique patient population.

In Korea, 25.6% of the perinatal medical care service areas are classified as having inconvenient access to maternal-fetal ICUs and NICUs, and there are mortality differences in these regions [24]. Currently, Korea’s medical system is centered around hospitals, resulting in poor access to medical care in rural areas. This is primarily due to a lack of medical service resources, such as transportation systems, a shortage of NICU beds, and the absence of professional medical staff [22,24]. Rehabilitation treatment is a hospital-centered medical service that is prone to regional disparities. Patients are willing to migrate between regions because of differences in the quality and quantity of medical services offered [23]. Indeed, the proportion of regional migration in the NICU group was significantly higher (3.8%) than that in the non-NICU group. This finding indicates that the level of medical services needed by NICU patients is not adequately provided within their local area. The Korean government is working to reduce patient mortality by increasing NICU-related medical expenses and national insurance coverage while also providing direct financial support to enhance NICU facilities, beds, and staffing in underserved urban areas [45]. As a result of these efforts, the number of NICU beds and facilities has increased; however, insufficient human resources and multidisciplinary support continue to be highlighted as issues [44,45]. In the future, the government must work to bridge regional gaps in rehabilitation services through policy initiatives. Efforts should focus on enhancing rehabilitation access in vulnerable areas and supporting local hospitals to provide high-quality medical staff, while adopting a multidisciplinary approach to rehabilitation services.

Results of this study indicated that, in the 6 months following the first rehabilitation session, the NICU group incurred total medical expenses that were more than six times greater than those of the non-NICU group. Previous studies have indicated that additional medical costs can vary from USD 10,055 to USD 15,440 due to potential morbidities following admission to the NICU [46]. Also, medical technology (oxygen supply, feeding tubes, tracheostomy, etc.) reportedly accounts for the largest proportion of medical expenses after NICU hospitalization, costing USD 33,276 in the first year after hospitalization [8]. In this study, medical costs in the NICU group were higher than those in the non-NICU group, even when analyzed by separating hospitalization and outpatient costs. Although medical costs were greater, the NICU group received significantly fewer treatments in the 6 months following their first rehabilitation session compared to the non-NICU group. This suggests that the potential morbidities and the costs associated with medical technology were greater than the expenses related to rehabilitation treatment. However, this cannot be definitively concluded from the results of this study alone. As it is essential to emphasize policy support and importance of neonatal rehabilitation, calculating the actual rehabilitation costs incurred during the early rehabilitation period for the NICU group should be investigated through follow-up studies.

Our study had several limitations. First, this study used claims data from the NHIS database, which is generally utilized for insurance reimbursements. Therefore, only the diagnosis, frequency of rehabilitation, and expenses are known, but clinical information, such as the severity of the disease and details of rehabilitation (e.g. intensity, treatment method, outcome), remain unknown. Furthermore, Factors such as the length of stay in the NICU, parents’ socioeconomic status, and the characteristics of the medical institution—such as the number of beds, medical staff, and available departments—were not included in the database. This oversight may result in confounding effects that have been overlooked. Therefore, future studies should aim to collect additional clinical data to minimize the impact of these confounding variables and highlight the importance of rehabilitation treatment for patients in the NICU. Second, because the total medical expenses were calculated based on what patients paid for all medical services provided during hospitalization or outpatient visits, it was difficult to interpret the differences between the two groups clearly. Third, this study was conducted using a single country’s national health insurance system, making it difficult to generalize the results to other countries’ medical service-related systems. In the case of Korea, which relies heavily on hospital-centered medical services, there are limitations to applying this model to a community-based medical system. The frequency with which patients use medical services and the associated costs will differ based on the coverage and out-of-pocket expenses of the nation’s health insurance system, and the influence of insurance systems on rehabilitation treatment rates should be further analyzed. Fourth, the results of this study analyze usage patterns, including the number and cost of rehabilitation treatments and the areas treated, but do not account for clinically relevant outcomes. Determining the optimal rehabilitation treatment strategy for NICU neonates is challenging due to the unknown long-term effects on neurodevelopment and functional recovery. Therefore, future studies may involve clinical measures to evaluate the impact of differing rehabilitation treatments (OT vs. PT, for example) between NICU and non-NICU groups on long-term developmental disabilities, which could inform strategies for NICU rehabilitation. Despite the findings being limited in generalizability for all newborns or at an international level, the rehabilitation status of patients with a history of NICU hospitalization was determined over a relatively long time period.

The rehabilitation of patients in the NICU group began at an earlier age than that of the non-NICU group; however, the number of rehabilitation sessions up to 2 years after the first rehabilitation session was less than that of the non-NICU group. The discrepancy between residence and area of first rehabilitation was significantly higher in the NICU group. Additionally, the total hospitalization medical expenses for 3 years after the first rehabilitation were significantly higher in the NICU group. These suggest that the rehabilitation status of patients in the NICU group is lacking in quantity and quality due to regional imbalances and systemic problems and that medical services related to disease treatment are used more often than rehabilitation. To address the needs of patients in the NICU, healthcare providers and the government must offer financial support for rehabilitation. This support should focus on areas experiencing personnel and facility shortages and aim to reduce overall medical costs.

## Supporting information

S1 Table Claim codes for treatments or procedures. (DOCX)

S2 Table Sensitivity analysis for propensity score matching. (DOCX)
