# Correction: Differences in rehabilitation for high-risk newborns: The impact of neonatal intensive care unit hospitalization

**DOI:** 10.1371/journal.pone.0335653

**Published:** 2025-10-29

**Authors:** Kyoung Yee Kang, Eun Sil Kang, Hye-Kang Park, Tae Mi Youk, Seung Been Hong, Ha Lim Lee

Tae Mi Youk should be included in the author byline. Tae Mi Youk should be listed as the fourth author, and affiliated with Research Institute, National Health Insurance Service Ilsan Hospital, Goyang, Gyeonggi-do, Republic of Korea. The contributions of this author are as follows: Data Curation, Formal Analysis, Investigation, Methodology, Validation and Writing – Review & Editing.

The correct citation is: Kang KY, Kang ES, Park H-K, Youk TM, Hong SB, Lee HL (2025) Differences in rehabilitation for high-risk newborns: The impact of neonatal intensive care unit hospitalization. PLoS One 20(5): e0322998. https://doi.org/10.1371/journal.pone.0322998.
