# Supplementary material for: Differences in rehabilitation for high-risk newborns: The impact of neonatal intensive care unit hospitalization
Source: PLoS One. 2025 May 9;20(5):e0322998. doi: 10.1371/journal.pone.0322998 (PMC12063853; doi:10.1371/journal.pone.0322998)
Supplement: S1 Table — (DOCX) [file pone.0322998.s001.docx]

**S1 Table. Claim codes for treatments or procedures**

| **Treatment/procedure** | **Health Insurance Review and Assessment claim codes** |
| --- | --- |
| Neonatal intensive care unit admission & nursing Fees | AJ111, AJ211, AJ311, AJ121, AJ221, AJ321, AJ101, AJ201, AJ301, AJ141, AJ241, AJ341, AJ131, AJ231, AJ151, AJ251, AJ144, AJ244, AJ161, AJ261, AJ331, AJ351 |
| Physical therapy | MM102, MM105, MM151, MM301, MM302, MM304 |
| Occupational therapy | MM112, MM113, MM114 |
| Whole body pool therapy | MM048 |
| Rehabilitative dysphagia therapy | MX141, MZ008 |
