# Supplementary material for: Differences in rehabilitation for high-risk newborns: The impact of neonatal intensive care unit hospitalization
Source: PLoS One. 2025 May 9;20(5):e0322998. doi: 10.1371/journal.pone.0322998 (PMC12063853; doi:10.1371/journal.pone.0322998)
Supplement: S2 Table — (DOCX) [file pone.0322998.s002.docx]

**S2 Table. Sensitivity analysis for PSMatching**

|  | within 6 months after the first rehabilitation session | | | | | | |
| --- | --- | --- | --- | --- | --- | --- | --- |
|  | Number of rehabilitation sessions per person | | | | | | |
|  | Non-NICU | | | NICU | | | p-value |
|  | N | Mean | (SD) | N | Mean | (SD) |  |
| Before Matching | 39,711 | 26.2 | (43.3) | 27,539 | 26.0 | (42.4) | 0.5394 |
| 1:1 PSMatching, caliper 0.2* | 16,626 | 30.2 | (47.6) | 16,626 | 21.9 | (39.9) | <.0001 |
| 1:1 PSMatching | 17,498 | 30.6 | (47.9) | 17,498 | 22.6 | (40.1) | <.0001 |
| 1:2 PSMatching | 11,850 | 24.7 | (40.8) | 5,925 | 18.0 | (35.8) | <.0001 |
| 1:3 PSMatching | 16,203 | 23.0 | (38.9) | 5,401 | 16.7 | (34.2) | <.0001 |
|  |  |  |  |  |  |  |  |
|  | within 6 months after the first rehabilitation session | | | | | | |
|  | Total medical expense(won) | | | | | | |
|  | Non-NICU | | | NICU | | | p-value |
|  | N | Mean | (SD) | N | Mean | (SD) |  |
| Before Matching | 39,711 | 1,932,774 | (8,124,331) | 27,539 | 11,085,412 | (22,284,464) | <.0001 |
| 1:1 PSMatching, caliper 0.2* | 16,626 | 1,868,516 | (7,094,255) | 16,626 | 11,348,940 | (22,603,784) | <.0001 |
| 1:1 PSMatching | 17,498 | 1,959,108 | (6,963,803) | 17,498 | 8,231,876 | (17,974,897) | <.0001 |
| 1:2 PSMatching | 11,850 | 1,857,053 | (9,132,646) | 5,925 | 8,267,504 | (19,518,768) | <.0001 |
| 1:3 PSMatching | 16,203 | 1,565,484 | (7,962,601) | 5,401 | 8,849,193 | (20,166,076) | <.0001 |

* The method used in this study.
